# Understanding nanocellulose chirality and structure–properties relationship at the single fibril level

**DOI:** 10.1038/ncomms8564

**Published:** 2015-06-25

**Authors:** Ivan Usov, Gustav Nyström, Jozef Adamcik, Stephan Handschin, Christina Schütz, Andreas Fall, Lennart Bergström, Raffaele Mezzenga

**Affiliations:** 1 Department of Health Science and Technology, ETH Zurich, Schmelzbergstrasse 9, LFO E23, Zurich 8092, Switzerland; 2 Department of Materials and Environmental Chemistry, Stockholm University, Svante Arrhenius väg 16C, Stockholm 10691, Sweden; 3 Wallenberg Wood Science Center, KTH, Teknikringen 56, Stockholm 10044, Sweden

## Abstract

Nanocellulose fibrils are ubiquitous in nature and nanotechnologies but their mesoscopic structural assembly is not yet fully understood. Here we study the structural features of rod-like cellulose nanoparticles on a single particle level, by applying statistical polymer physics concepts on electron and atomic force microscopy images, and we assess their physical properties via quantitative nanomechanical mapping. We show evidence of right-handed chirality, observed on both bundles and on single fibrils. Statistical analysis of contours from microscopy images shows a non-Gaussian kink angle distribution. This is inconsistent with a structure consisting of alternating amorphous and crystalline domains along the contour and supports process-induced kink formation. The intrinsic mechanical properties of nanocellulose are extracted from nanoindentation and persistence length method for transversal and longitudinal directions, respectively. The structural analysis is pushed to the level of single cellulose polymer chains, and their smallest associated unit with a proposed 2 × 2 chain-packing arrangement.

## Abstract (web summary)

Cellulose is a material found in many different biological systems, but the fine structure at the single-molecule level is still being assessed. Here, the authors present high-resolution imaging of cellulose structures at the single particle level, finding evidence of chirality in bundles and fibrils.

## Graphical abstract

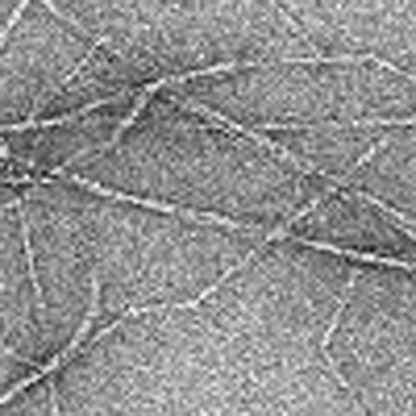

In nature, cellulose, the major load-bearing structure in all living plants, shows complex superstructures, chirality and chiral inversions over different length scales^1^,^2^. This fascinating structural behaviour, in combination with a high specific strength and stiffness, has inspired work to engineer cellulose materials with tailored mechanical and optical properties^3^,^4^. Over the last decades, elongated rod-like cellulose nanoparticles, shorter cellulose nanocrystals (CNCs) and longer cellulose nanofibrils (CNF), have been used to form chiral nematic liquid crystals^5^, aerogels^6^, photonic^7^ and inorganic hybrid materials^8^. Even though these materials have impressive properties and show great potential for a broad range of applications, the fine structure of their nanocellulose components has not yet been fully elucidated^9^. Only through a detailed knowledge of the chirality and the structure of the smallest nanocellulose building blocks, new strategies for advanced bottom-up nanotechnologies and assembly of new helical metamaterials may become available.

On the atomic length scale, high-resolution neutron and X-ray scattering data have revealed the crystalline structure of cellulose down to the exact atomic positions of the polymers in the unit cell^10^. How CNC arrange in chiral nematic liquid crystalline phases^11^,^12^,^13^ and how CNF arrange in films^14^, aerogels^15^ or foams^16^ is also well characterized. In between these two length scales, however, a gap remains, where the structure and assembly of nanocellulose fibrils are not yet fully understood. Given the hierarchical nature of cellulose, an investigation of this intermediate length scales may reveal important structural information, improve the fundamental understanding and pave the way to new strategies in materials assembly.

Traditional nanocellulose research relies on the disintegration of a cellulosic raw material into its smallest possible components (CNC and CNF) and the subsequent re-assembly of these building blocks into new materials. The harsh chemical and mechanical treatments used in the disintegration process may have a large influence on the final structural properties of the cellulose nanoparticles^17^. This is often overlooked and only a few detailed experimental investigations have been performed^18^,^19^.

Previous experimental microscopy and scattering work have allowed resolving average particle dimensions^20^, indirect (from neutron scattering)^21^ and direct evidence (from fibril bundles)^22^,^23^ of particle twist, as well as local mechanical properties with peak force quantitative nanomechanical mapping (PF-QNM)^24^,^25^. Simulations have confirmed a twisted structure at equilibrium^26^,^27^, and also estimated single particle mechanical properties similar to the measured values^28^. However, issues related to the quantification and origin of the CNF conformations remain elusive and the fundamental question of the (in)solubility of cellulose in water has recently become a matter of intense debate^29^. With the rapid development of experimental techniques, new and more detailed structural information becomes available, allowing these outstanding fundamental questions to be revisited and conclusively assessed.

In this work, state-of-the-art atomic force, cryogenic scanning electron and transmission electron microscopy (AFM, Cryo-SEM and TEM, respectively) are combined with advanced statistical analysis and concepts from polymer physics to investigate three different types of nanocellulose, TEMPO(2,2,6,6-tetramethylpiperidine-1-oxyl)-mediated oxidized wood cellulose nanofibrils (W-CNF), wood cellulose nanocrystals (W-CNC) and sulfuric acid hydrolyzed bacterial cellulose nanocrystals (B-CNC). All samples show clear evidence of a right-handed chirality both at the level of bundles of fibrils and on the individual fibril level (W-CNF). Deeper investigation of one of the nanocellulose families (W-CNF) reveals detailed information on particle dimensions and provides compelling evidence for multiple levels of substructures, down to the single cellulose chain. From the statistical analysis, the persistence length of the W-CNF is extracted, and indirectly, its intrinsic rigidity in the longitudinal direction, while the direct measurements of the Young's moduli obtained using PF-QNM characterize mechanical properties of the nanocellulose samples in the transversal plane. Most importantly, the detailed statistical investigation of the morphology of the W-CNF provides convincing evidence that the commonly accepted model of CNF structure as built of alternating regions of crystalline and amorphous cellulose domains along the fibril length^9^,^30^,^31^ cannot explain the presence of kinks.

## Results

### Overview of the nanocellulose systems

**Figure 1:**
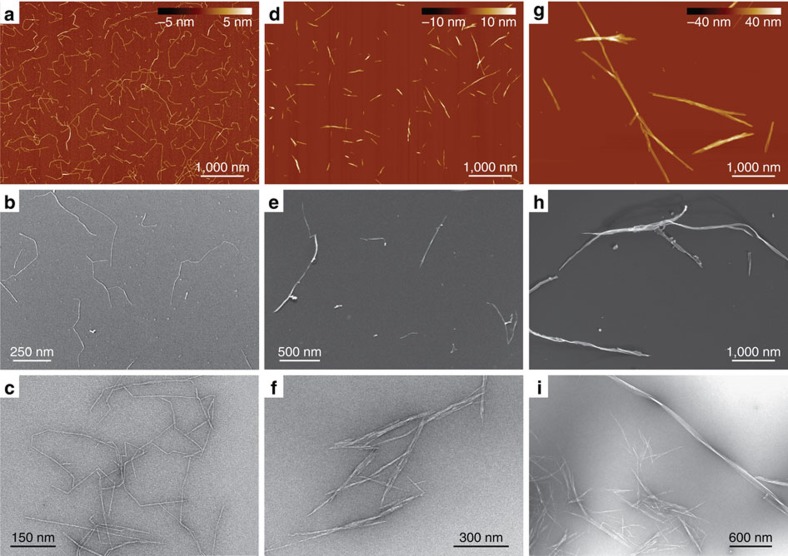
Overview microscopy images of the nanocellulose samples. (**a**–**c**) TEMPO-mediated oxidized W-CNF, (**d**–**f**) W-CNC and (**g**–**i**) B-CNC samples via AFM (**a**,**d**,**g**) Cryo-SEM (**b**,**e**,**h**) and TEM (**c**,**f**,**i**). All AFM images have the same magnification in order to provide a direct comparison between nanocellulose particles.

The low-magnification images of the three different nanocellulose systems (W-CNF, W-CNC and B-CNC) via three different microscopy techniques (AFM, Cryo-SEM and TEM) are shown in Fig. 1. On the basis of the initial visual inspection we provide a description of their morphology and typical structural parameters. In the W-CNF (Fig. 1a–c), CNFs have a relatively constant cross-section with average widths in the range of ∼2−3 nm and lengths ∼0.1−1 μm. One particular feature of this sample is that almost every nanofibril has sharp bends between otherwise straight segments, a feature hereinafter referred to as kinks. Moreover, these nanofibrils can sometimes still be observed to be assembled in fibril bundles, despite the strong mechanical treatment applied during the homogenization process for sample preparation. The W-CNC sample (Fig. 1d–f) is represented by rather straight particles that have a large variation in cross-section along their contours. They can be described as large multistranded bundles of laterally assembled CNFs. Their average widths are in the range of ∼3−30 nm, but the lengths are similar to the W-CNF, that is, ∼0.1−1 μm. In rare occasions it is possible to observe thin segments with kinks, a signature of the W-CNF precursor samples, indicating that the hydrolysis was not fully completed (Supplementary Fig. 1). For the B-CNC (Fig. 1g–i), the observed nanofibrils have typical lengths of ∼1–5 μm and irregular widths in the range of ∼10−50 nm. The features of nanocellulose systems are consistent within all distinct microscopy techniques used in this study, which confirms that any possible artefact associated with either the microscopy technique or sample preparation procedure is below the interpretable resolution.

During the preparation of the nanocellulose samples, ionizable groups are introduced (-COOH for W-CNF and W-CNC, and -SO_3_H for B-CNC) that electrostatically improve the stability of the particles in dispersion. The charge density of W-CNC and B-CNC was measured with polyelectrolyte titration, yielding gravimetrically normalized values of 0.4 mmol g^−1^ (W-CNC) and 0.03 mmol g^−1^ (B-CNC). Because the charge density is not expected to be affected by the hydrolysis treatment^32^, we assign a charge density of 0.4 mmol g^−1^ also for the W-CNF sample. On the basis of the microscopy images, we find that there is a larger fraction of individual particles of W-CNF compared with W-CNC. The better colloidal stability of W-CNF may be related to a steric hindrance effect due to the kinks preventing neighbouring fibrils to come into close contact and form bundles. The stronger tendency for B-CNC compared with W-CNC to form bundles is probably related to the relatively low charge density of B-CNC. It should also be noted that the pH of the W-CNC and B-CNC samples is drastically decreased during hydrolysis, causing a reduction in the charge density of the CNC particles, resulting in the formation of bundles and aggregates. It is possible that not all of these aggregates become fully redispersed when the pH is readjusted.

### All types of nanocellulose possess a right-handed chirality

**Figure 2:**
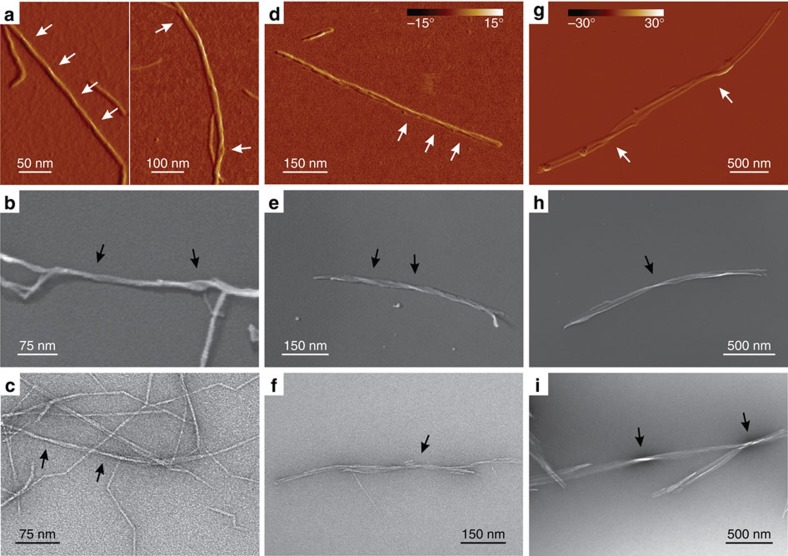
All samples show right-handed twisting. (**a**–**c**) TEMPO-mediated oxidized W-CNF, (**d**–**f**) W-CNC and (**g**–**i**) B-CNC. The images are acquired from the AFM amplitude channel (**a**, left) and phase channel (**a**, right and **d**,**g**), Cryo-SEM (**b**,**e**,**h**) and TEM (**c**,**f**,**i**). The arrows point to regions along fibril or crystal contours, where it is possible to detect a right-handed twisting. The corresponding AFM height maps for the images **a**,**d**,**g** are shown in Supplementary Fig. 3. The colour bar in **g** also applies to **a**.

The magnified microscopy images reveal that all cellulose fibrils and crystals with observable chirality are right-handed (Fig. 2). For the W-CNF (Fig. 2a–c), right-handed chirality is observed both on the single fibril level (Fig. 2a, left and Supplementary Fig. 2) and for bundles of fibrils (Fig. 2a, right). We note that a twist in amplitude measurements is observable only if the local microfibril cross-section has distinct corners—for cylindrical fibrils this would be invisible. For the CNC particles, chirality observations were only possible on crystal bundles, since isolated CNCs with constant and uniform cross-section could not be observed.

The presence of the right-handed twist along cellulose nanoparticles and bundles of nanoparticles is not routinely observable (5–10% from hundreds of visualized particles); however, when a twist is observable, it is always right-handed. This relatively low amount of twisted particles in W-CNF could be explained by a finite AFM resolution, which is approaching the limit to detect these features. For W-CNC and B-CNC, the twisting normally occurs in bundles with high structural organization of single particles, but could be hidden in the case of loosely packed aggregates.

These observations are in line with previous experimental^22^,^23^ and theoretical^26^,^27^ studies, supporting a right-handed chirality. However, the experimental results presented to date, rely on higher-order structures of fibril aggregates that are in μm-length scale. This distinction is important since there are different examples in nature where chirality inversion occurs over different length scales^33^ and cellulose may follow the same trends. For example, the observed cholesteric liquid crystal ordering of CNCs is left-handed^34^ and the tracheary elements, responsible for transporting water in most plants, are reinforced by left-handed helical cellulose thickenings^2^. In contrast to these observations of higher-order structures, the results presented here establish that the single CNFs with observable chirality always bear a right-handed twisting. Because also the observed chirality of the fibril bundles is right-handed, this indicates that there is no chiral inversion on this length scale.

Recent studies based on density functional theory of right-handed hard helices indicate that the way chirality transfers from the building block to the chiral nematic phase is non-trivial and depends at least on two different parameters, entropy (excluded volume of left-/right-handed particle pairs) and thermodynamics (volume fraction of rods)^35^. This suggests that a chirality inversion from a right-handed particle to a left-handed chiral nematic phase may be expected, provided that the concentration of rods in the chiral nematic is in the right range^35^. Thus, the results at the single fibril and small bundle level given here may provide the necessary information to bridge the gap between theoretical models and experimental results in nanocellulose cholesteric nematic phases.

### PF-QNM of the nanocellulose samples

**Figure 3:**
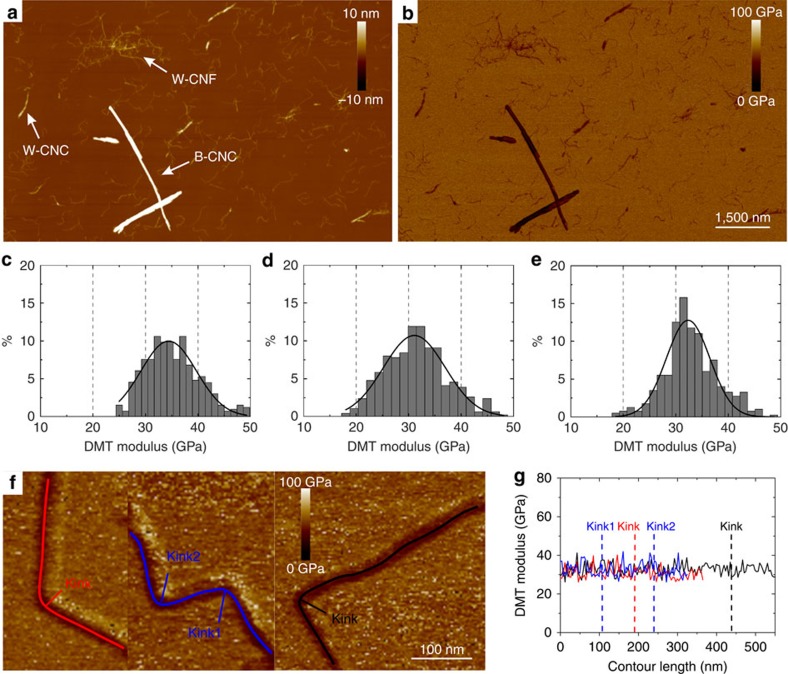
Peak force quantitative nanomechanical mapping. (**a**) AFM height channel visualizing the composition of all nanocellulose samples mixed in 1:1:1 proportion. The nanocellulose particles are distinguished and labelled according to their morphological features. (**b**) The corresponding PF-QNM map. The scale bar applies to both **a**,**b** panels. (**c**–**e**) DMT modulus distributions measured on particles representing (**c**) W-CNF, (**d**) W-CNC and (**e**) B-CNC. (**f**) PF-QNM images of W-CNFs and their profiles represented by red, blue and black lines. (**g**) Mechanical properties along the profiles of fibrils shown in **f** and the corresponding kink positions.

The mechanical properties of cellulose in the transversal direction were probed using PF-QNM^36^. Previously, this method has been successfully used for systems of amyloid fibrils^37^,^38^ that have similar dimensions to cellulose fibrils. Figure 3a shows an AFM image with the composition of all three types of nanocellulose particles: W-CNF, W-CNC and B-CNC, mixed in 1:1:1 proportion, with the corresponding PF-QNM map depicted in Fig. 3b. The Young's modulus values, *E*_QNM_, were estimated according to the Derjaguin–Mueller–Toporov (DMT) model, and were collected along the contours of each cellulose type to obtain the corresponding modulus distributions (Fig. 3c–e). We find that the Young's moduli lay in the range of 20–50 GPa for all three types of nanocellulose particles, and are in good agreement to previously measured moduli from PF-QNM on nanocellulose (6–50 GPa; refs 24, 25). The Gaussian fits of these distributions with parameters *μ* and *σ* (mean value and s.d.) result in the following elastic moduli (*μ*±*σ*): 34.4±5.3 GPa for W-CNF (Fig. 3c), 31.1±5.9 GPa for W-CNC (Fig. 3d) and 32.3±4.1 GPa for B-CNC (Fig. 3e). Moreover, we note that the PF-QNM measurements yield remarkably close values of Young's moduli for all three samples investigated. Since the AFM sample preparation conditions allow for a qualitative side-by-side comparison of the samples, these results indicate that the mechanical properties of the samples do not depend on the nanocellulose-processing conditions.

### Tracking and statistical analysis of W-CNF

The TEMPO-mediated oxidized W-CNF system is of particular interest for the application of statistical analysis since in comparison with the other nanocellulose systems, it has rather uniform contour shapes. The statistical analysis of the nanofibril contours provides unique insight on the nature of their kinks, and moreover, it allows an estimation of the Young's modulus value of rigid segments via the persistence length approach^39^. Furthermore, the basic morphological parameters such as length and height distributions allow elucidating the packing model of single cellulose polymer chains within the nanocellulose fibril.

**Figure 4:**
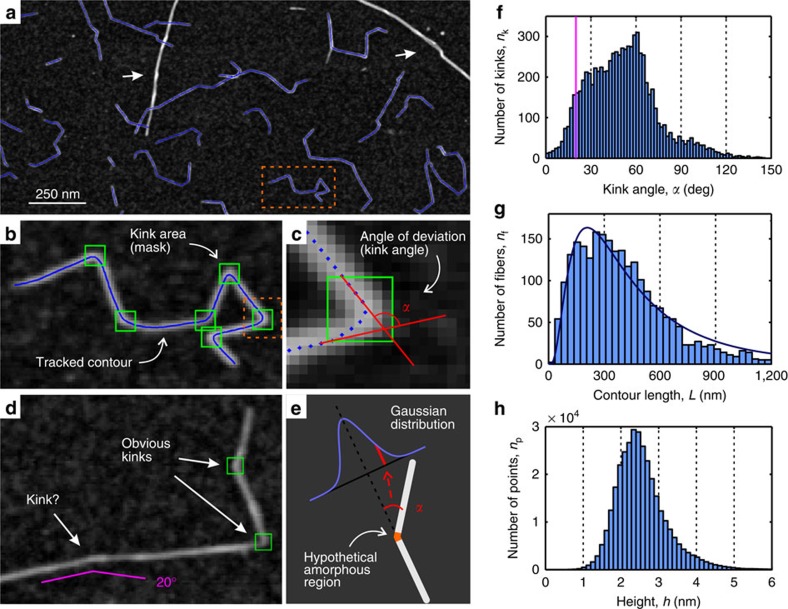
Tracking procedure of W-CNF and results of statistical analysis on fibril contours. (**a**) AFM image with tracked contours (blue curves). Objects that are considered to be bundles of CNF were discarded from tracking (examples are pointed by white arrows). (**b**) Magnified region of the AFM image from **a** containing one tracked contour. The green frames are special masks, placed manually, allowing tracking of the contours with heterogeneous flexibility. In the statistical analysis these areas were considered to represent the vicinities of kinks. (**c**) Magnified region of the AFM image from **b** showing the particular mask area with the contour represented by its points. Two segments of the contour that enter or exit the kink area define an angle of contour deviation (kink angle) *α*. (**d**) Example of CNF with two obvious kinks and one uncertain case. It is non-trivial to state whether this is a kink or elastic bending due to thermal fluctuations. An example of the 20° angle is shown as a guide to eye. (**e**) The kink angle distribution should have a Gaussian shape in case of hypothetical amorphous regions corresponding to kinks because of equal probability to bend in both directions. (**f**) Kink angle distribution of 2,380 tracked CNFs. The bins in the region below 20° (violet line corresponds to this cutoff value) can lack some counts because of the manual threshold of kink angle assignments. (**g**) Contour length distribution fitted with normalized density function of the log-normal distribution with parameters *μ*=5.94 and *σ*=0.77. (**h**) Height distribution of all points along all tracked contours. The most probable height value is 2.35 nm.

We have used a tracking procedure to obtain coordinates of the CNF contours (Fig. 4a, blue lines) similar to the one used in previous lines of work on amyloid fibrils^33^,^40^, carrageenan polysaccharide polymers^41^ or carbon nanotubes^42^. The coordinates of 2,380 fibrils were acquired from AFM images that covers 450-μm^2^ area of mica substrate, using a specially designed in-house software written in MATLAB (see Methods)^43^. Objects that were considered to be bundles of fibrils with distinctively larger diameters were, whenever possible, discarded from the analysis. The positions of the kinks were determined manually using special masks (Fig. 4b, green boxes). Two segments of a contour that are crossing a mask element (entering and exiting segments) define a contour angle (Fig. 4c), which is also referred as a kink angle. One important note to mention is that it is difficult to distinguish between very small kink angles (below 20°) and random thermal fluctuation in the AFM images. For example, Fig. 4d depicts a fibril with two obvious kinks that are unambiguously determined and one particular place with a hint of a kink only. The threshold for observable kinks does affect the results, but in a predictive manner.

It is well known that CNF consists of cellulose chains with different degrees of order, from highly crystalline arrangements to a slightly perturbed distribution of the chains. Two models have been proposed to describe the distribution of the disordered and ordered regions in CNF^44^. In the first model, the less-ordered chains, often referred to as amorphous chains, are distributed between crystalline regions of chains along the fibril direction (analogous to semicrystalline polymers)^45^. In the second model, the less-ordered chains are located towards the surface of fibrils with a crystalline core of chains^46^. The former model is commonly invoked as a rationale for CNC particle preparation, as hydrolysis is believed to primarily dissolve the amorphous regions of cellulose chains, leaving only the short, crystalline particles. However, if the regions between the crystalline parts are randomly packed arrangements of moderately oriented chains—amorphous regions to a good approximation—we would expect a Gaussian distribution of the kink angles because of equal probability to bend in both directions, and, thus, leading to a Gaussian distribution of the excess, that is, the observable kink angle, as in general random walk statistics. We note that a Gaussian distribution of bending angles is also expected on the basis of the worm-like chain formalism. It is clear that the observed peak in the kinks distribution at 60° does not support the first model, and the alternative model with less-ordered surface chains may possibly be more appropriate to describe the cellulose polymer-chain arrangement in CNF.

In order to estimate the influence of the contact with the substrate surface on the distribution of kink angles, the analogical analysis was conducted for images obtained using Cryo-SEM and AFM on graphite substrate (Supplementary Fig. 4). The number of traced fibrils was 100 and 120 for Cryo-SEM and AFM on graphite correspondingly; hence, the statistics is 20 times less accurate than the one reported in the main text, yet, the same conclusion is supported in both cases.

We further note that the distribution of kink angles given in Fig. 4f accounts for kinks observed in fibrils of polydisperse cross-sections. This may imply, in principle, that each individual fibril can contribute to the final statistic with its own kink angle distribution. In such an eventuality, by maintaining the assumption of kink angles originating from amorphous domains, the final kink angle distribution would then be the weighted sum of individual (symmetric) Gaussian distributions reflecting different angle probabilities for different fibril thicknesses, resulting into a non-Gaussian, yet fully symmetric, final distribution of kink angles. It is therefore the non-Gaussian and non-symmetric nature of the distribution given in Fig. 4f, which allows ruling out amorphous domains as a cause of the presence of kinks, independently of the cross-section distribution of the nanocellulose fibrils considered in the statistics.

Additional support to the conclusions drawn from the kink angle distribution analysis, ruling out the presence of ‘softer' amorphous zone regions alternating to stiffer crystalline ones, is given by the lack of significant changes in Young's moduli measured using PF-QNM in correspondence of the kink regions, which would be expected if these regions were amorphous (see Fig. 3f,g). The crystalline core-amorphous shell model is also in line with previous NMR studies^46^,^47^,^48^, as well as the recent detailed experimental study that combined small-angle neutron scattering, wide-angle X-ray scattering, NMR and Fourier transform infrared spectroscopy^20^. Our results are therefore suggesting that the observed kinks may result from the mechanical treatment during the sample preparation^19^ and not from the presence of amorphous regions on the fibril contour.

Many size measures in nature tend to have a log-normal distribution, for example, the lengths of inert appendages (hair, claws and nails) in biology^49^, or the lengths of amyloid fibrils^50^; here we show that the length of CNFs follows the same type of distribution. The log-normal distribution has the following probability density function *f*(*L*; *μ*, *σ*):


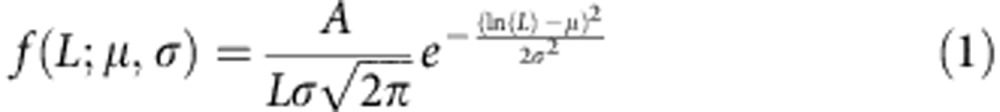


where *L* is the total contour length of the fibrils, *μ* and *σ* are the mean value and the s.d. of the length's natural logarithm, respectively, and *A* is a distribution normalizing constant. The length distribution of W-CNF is shown in Fig. 4g with a log-normal distribution fit. Parameters of the fit are as follows: *μ*=5.94±0.08 and *σ*=0.77±0.06, which corresponds to the average length 〈*L*〉=511 nm via the well-known relation 
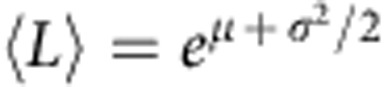
.

**Figure 5:**
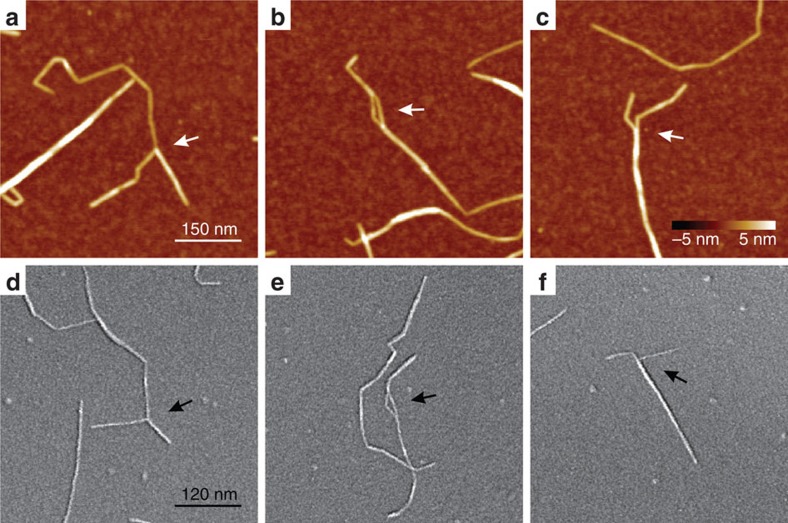
Examples of splitting of W-CNF. The splitting event might occur at the end of W-CNF (**a**,**c**,**d**,**f**), as well as along the contour (**b**,**e**). The arrows point towards regions of interest. The images are obtained by (**a**–**c**) AFM and (**d**–**f**) Cryo-SEM. The scale bar in **a** and the colour bar in **c** apply to all AFM images (**a**–**c**). The scale bar in **d** applies to all Cryo-SEM images (**d**–**f**).

The height distribution of W-CNF is shown in Fig. 4h. The height of the rigid segments does not differ from the height in the vicinities of kinks (Supplementary Fig. 5) and is estimated to be in the range *h*=(1.9–2.7) nm, with the most probable height value 〈*h*〉=2.35 nm, which corresponds to the average diameter of fibrils. The broad height distribution of the W-CNF peak can be attributed to the possibility of fibrils to split and thus have various sizes and packing models. Some representative examples of fibril-splitting are shown in Fig. 5.

### Persistence length and second moment of area of the W-CNF

The persistence length *λ* is an essential characteristic of polymers, or in general fibrillar-like objects, and is directly related to the mechanical properties on a longitudinal inflection. It is formally defined as the length over which an angular correlation in the tangent direction to a fibril contour is decreased on average by *e* times in three-dimensional space^51^. Following this definition, the bond correlation function (BCF) is the most common way to evaluate the persistence length and in two-dimensional space it has the following form^52^: 〈cos*θ*〉=*e*^−*l*/2*λ*^, where *θ* is the angle between tangent directions of any two segments along a fibril contour. Another useful method of the persistence length estimation is to evaluate the mean-squared end-to-end distance (MSED) between contour segments of a fibril, which for a worm-like chain model has a following theoretical dependence on the internal contour length *l* in two-dimensional space^53^: 〈*R*^2^〉=4*λ*[*l*–2*λ*(1–*e*^−*l*/2*λ*^)], where *R* is the direct distance between any pair of segments along a fibril contour. A different method that can be successfully applied to very stiff fibrillar-like objects, for which at internal contour length is smaller than the corresponding persistence length (*l*<*λ*), is the mean-squared midpoint displacement (MSMD). The equation, describing the behaviour of an arc midpoint deviation, is derived with the assumption that this deviation is small in comparison with the corresponding internal contour length (|*u*_*x*_|<<*l*) and thus has the form^54^ 
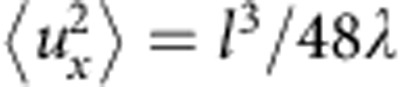
, where 
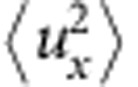
 is the midpoint mean-squared displacement between any pair of segments along a fibril contour.

**Figure 6:**
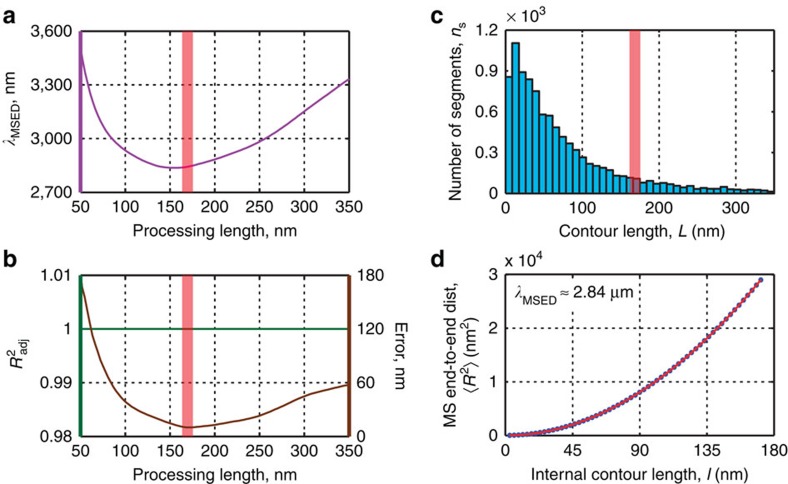
Length distribution of W-CNF rigid segments and estimation of their persistence length. (**a**) Persistence length *λ*_MSED_ calculated via the MSED method versus the processing length. (**b**) Adjusted coefficient of determination (goodness of fit) 
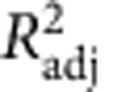
 and fitting error versus the processing length. (**c**) Length distribution of W-CNF rigid segments. The red vertical lines in **a–c** correspond to the processing length 170 nm at which the fitting error is minimal. (**d**) MSED versus internal contour length fit at the distance with the minimal fitting error. The resulting persistence length is *λ*_MSED_=2.84 μm.

Because the kinks of the W-CNF may originate from the mechanical treatment, the deformation in these areas did go beyond the elastic limit and, thus, cannot represent elastic properties of the material on axial bending. We thus divide the W-CNFs using mask elements as split points and discard areas inside them to avoid kinks. Hence, in the resulting data, individual contours correspond only to rigid segments. The value of the persistence length obtained by the end-to-end distance versus internal contour length is *λ*_MSED_=2.84 μm (Fig. 6). Supplementary Fig. 6 shows the analogue estimations of persistence length of W-CNF rigid segments via BCF and MSMD. The resulting values are close: *λ*_BCF_=2.54 μm and *λ*_MSMD_=2.49 μm. However, the relative errors and fitting quality provided by the end-to-end distance method is better than by the other two approaches (Supplementary Fig. 6b,e). We use only the data of the rigid segments in the Young's modulus calculation, but because of the mentioned inability to identify all kinks, the persistence length may be slightly underestimated.

The persistence length is related to the bending rigidity, *EI*, of the fibrillar-like object: *EI*=*λk*_B_*T*, where *k*_B_ is the Boltzmann constant and *T* is the temperature^55^. As we reported before, the height and thus the diameter of the cellulose rigid segments can be estimated to be *h*=(1.9–2.7) nm. Here we assume a round cross-section for CNFs and, taking into account errors on the height values, the area moment of inertia lays in the range *I*=*πh*^4^/64=(0.64–2.61) nm^4^. Together with *k*_B_*T*=4.14 × 10^6 ^Pa nm^3^, the Young's modulus is found to be *E*_*λ*_=*λk*_B_*T*/*I*=4.5–18.4 GPa. The deviation from the average height has the highest impact on the elastic modulus value *E*_*λ*_ due to the power 4 dependence of the area moment of inertia *I* (ref. 56); therefore, we neglect all other possible sources of errors in this calculation. Because of the underestimation of the persistence length as a result of lost kink counts at low angles, this value is also underestimated. Furthermore, nanocellulose, as many other types of natural fibrils, is known to possess anisotropy in the continuum mechanical property tensor and therefore, the elastic behaviour in the axial and radial directions are expected to differ. Thus, while in other natural fibrous systems, small differences between the nanoindentation measurements and the results of statistical analysis of fibril bending may arise from slightly different elastic moduli on longitudinal bending *E*_*λ*_ and on transversal bending *E*_QNM_ (refs 33, 39), in the case of nanocellulose, this deviation may become more significant^9^.

### Observation of single cellulose polymer chains

**Figure 7:**
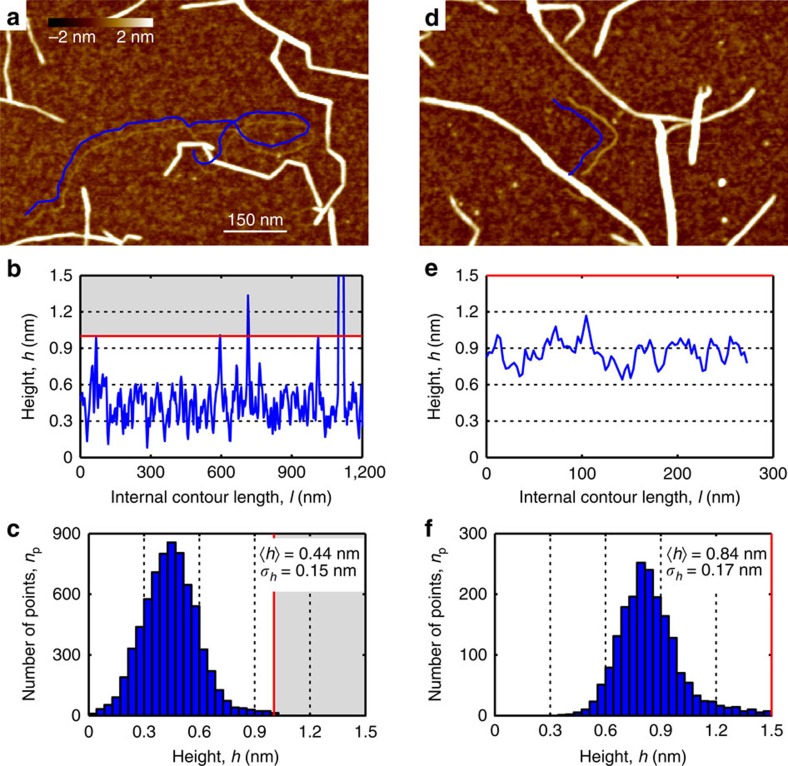
Observation of single cellulose chain and nanofibril with a 2 × 2 chain-packing structure. (**a**–**c**) The single polymer chain and (**d**–**f**) nanofibril with polymer chains composed in a possible 2 × 2 chain-packing structure. (**a**,**d**) AFM images with the tracked contours represented by blue lines that are slightly shifted for better visualization. (**b**,**e**) Height profiles along the tracked contours from **a**,**d**, respectively. (**c**,**f**) Height distributions of points along 20 tracked single cellulose polymer chains with the average height *h*=0.44 nm and s.d. *σ*_*h*_=0.15 nm, and 2 × 2 cellulose nanofibril with the average height *h*=0.84 nm and s.d. *σ*_*h*_=0.17 nm. Cutoffs at 1 nm (**b**,**c**) and 1.5 nm (**e**,**f**) were introduced to discard height data from chain-fibril crossings.

In rare occasions it is possible to observe very thin, possibly single cellulose polymer chains (Fig. 7a). This observation is in agreement with previous observations of molecularly thin sections along CNF contours^57^,^58^. In contrast to those findings, however, our data show fully flexible particles indicating that the observed objects are single polymer chains rather than single crystalline layers of cellulose chains, as suggested in (ref. 58). A longitudinal section is shown in Fig. 7b, while the height distribution of 20 tracked fibrils is presented in Fig. 7c (average height 0.44±0.15 nm). Owing to crossings with other thicker fibrils and the polymer overlapping with itself, we set an upper limit for possible real height values at 1 nm. Similar objects could be detected also in the W-CNC sample (Supplementary Fig. 7a–c). The root mean-squared surface roughness of the (3-Aminopropyl)triethoxysilane-modified mica surface between cellulose particles is 0.19 nm, which explains the relatively high variation in the longitudinal height profile. The obtained height value is in good agreement with the dimensions of the cellulose chain within the cellulose Iβ crystal structure with a monoclinic unit cell and base lattice parameters *a*≅0.78 nm and *b*≅0.82 nm (refs 10, 26). Taking into account the most probable height value for the majority of the tracked W-CNFs (2.35 nm), this suggests that a 4 × 4 cellulose chain-packing arrangement is the dominating structure. However, the height distribution broadness of W-CNF (Fig. 4h), together with evidence of splitting also supports the presence of *n* × *m* structures, where *n* and *m* could possibly be equal to 3, 4, 5 and 6. The structure of cellulose fibrils in wood, based on scattering measurements, was reported to contain 24 polymer chains and favoured a ‘rectangular' model^20^, which is close in numbers to what we have derived in this study. Furthermore, in very rare occasions we detected thin W-CNF in which the cellulose polymer chains possibly packed in a 2 × 2 chain-packing structure (Fig. 7d). The longitudinal section and the height distribution are shown in Fig. 7e,f. The average height for these objects is almost double in comparison with single polymer chains and equals to 0.84 nm (Fig. 7f). By analogy, we set up an upper limit at 1.5 nm for the possible height values. Supplementary Fig. 7d depicts both single and 2 × 2 cellulose polymer chains forming a remarkable network with a well-defined entanglement centre. Such structures could possibly originate from peeling off single cellulose chains during the harsh mechanical CNF preparation process.

Finally, we calculated the persistence length for single cellulose polymer chains adsorbed on mica via the BCF and the MSED methods. The MSMD method cannot be suitably used here since the assumption that the persistence length should be much larger than the contour length—as we discussed before—is not justified in the present case. The resulting values are: *λ*_BCF_=65 nm (Supplementary Fig. 8a–c) and *λ*_MSED_=63 nm (Supplementary Fig. 8d–f), respectively. These values are much larger than the theoretical and simulation predictions of the ‘bare' persistence length, reported to be in the range of 5–15 nm (ref. 59). We explain this discrepancy with the electrostatic repulsion between charged monomers along polymer chains and employ the Odijk–Skolnick–Fixman (OSF) theory^60^,^61^ to rationalize this observation. Within the framework of the OSF theory the total persistence length can be represented as a sum of ‘bare' (*λ*_0_) and electrostatic (*λ*_OSF_) components: *λ*=*λ*_0_+*λ*_OSF_, where 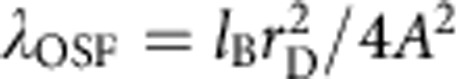 (ref. 62), *r*_D_ is the Debye length, *l*_B_=0.71 nm is the Bjerrum length and *A* is the distance between two neighbouring charged units. The charge density of 0.4 mmol g^−1^ was measured at pH 10, but the final value, at which the AFM samples were prepared, was pH 5.65. By taking this pH change into account as well as the contribution from the counterions (*I*_cI_=4 × 10^−6 ^M), the ionic strength is estimated to *I*=6 × 10^−6 ^M. Using the previously established approach^63^ we calculate the degree of dissociation *β*=0.08, which leads to an effective charge density of 0.032 mmol g^−1^. Assuming the distribution of charges only on the surface of particles, with average dimensions obtained from the statistical analysis, an average distance between charges of *A*≅7.7 nm is found. Under these conditions of ionic strength, the Debye length *r*_D_ can be estimated to be *r*_D_≅122 nm, and the resulting *λ*_OSF_≅44.7 nm, leading to a total expected persistence length of ∼50–60 nm, which is in excellent agreement with the experimental results (*λ*_BC_=65 nm; *λ*_MSED_=63 nm), providing a solid basis for the understanding of the large observed persistence length of individual nanocellulose chains. In order to test whether counterion condensation plays a role in the final observable persistence length, we perform a quick Oosawa–Manning counterion condensation check. The condensation of counterions occurs when *l*_B_*ρ*>1, where *ρ* is the linear charge density (*ρ*=1/*A*=0.13 nm^−1^). In the present case *l*_B_*ρ*≈0.09, suggesting that counterion condensation can be discarded.

## Discussion

In the present work we provide a comprehensive and consistent structural description over multiple length scales of nanocellulose of different origin and pretreatment: TEMPO-mediated oxidized W-CNF, W-CNC and B-CNC. We find that all types of nanocellulose fibrils and crystals with an observable twisting along the contour possess a right-handed chirality. The right-handed chirality was detected on the level of fibril bundles in all systems and on the single fibril level in W-CNF. By a statistical analysis of kink angle distribution, we conclude from the absence of a Gaussian distribution of kink angles that the kinks present in W-CNF are not a result of alternating amorphous and crystalline domains, as previously proposed in literature^64^,^65^,^66^. Rather they may originate from the processing conditions used in the preparation of the nanofibrils. PF-QNM was used to probe mechanical properties of the nanocellulose in the transversal direction, showing values of Young's moduli, *E*_QNM_, in the range of 20–50 GPa. Using the persistence length *λ* method, Young's moduli in the longitudinal direction were extracted in the *E*_*λ*_=4.5–18.4 GPa range, although this range can be underestimated because of the influence of undetected kinks with small angle of deviations. Finally, we have described the statistics of single free cellulose polymer chains, detected in both W-CNF and W-CNC and extracted a persistence length in the range *λ*=63–65 nm. The discrepancy between ‘bare' persistence length *λ*_0_=5–15 nm and the observable persistence length originates from electrostatic repulsion and can be rationally explained by the OSF theory.

## Methods

### Preparation of TEMPO-mediated oxidized W-CNF

The TEMPO-mediated oxidation of wood pulp was performed according to the procedure introduced in ref. 67. The soft-wood pulp was first treated in a phosphate buffer at pH 6.8 at 60 °C. The desired amount of sodium chlorite, TEMPO and sodium hypochlorite was added and the dispersion was stirred for 2 h and 20 min, after which the pulp was washed with deionized water and collected with vacuum filtration. The TEMPO-mediated oxidized material was dispersed in deionized water and disintegrated by homogenization with a Microfluidizer M-110 EH (Microfluidics, USA), in a sequence consisting of four passes through the microchannels with diameters of 400–200 μm at a pressure of 900 bar and for four passes through the microchannels with diameters of 200–100 μm at 1,500 bar. The resulting suspension of TEMPO-mediated W-CNFs was first sonicated for 10 min using a 13-mm-wide titanium probe at an output power of 70% (Vibra-Cell VC 750, Sonics, USA), followed by centrifugation for 60 min at 4000, g. The W-CNF dispersion was diluted to 0.001 w/w% with MilliQ water and shaken thoroughly.

### Preparation of W-CNC

W-CNCs were prepared by hydrochloric acid hydrolysis of the TEMPO-mediated oxidized CNFs (W-CNF) according to the procedure described in (ref. 32). Hydrochloric acid to a final concentration of 2.5 M was added to a dispersion of 100 g of a 1 w/w% (dry weight basis) W-CNF gel diluted with 316 ml deionized water and heated to 105 °C for 6 h. The reaction was quenched by dilution with the fivefold amount of deionized water. The hydrolysed material was washed with deionized water, collected by centrifugation for 10 min at 4000, g and dialysed for 5 days against deionized water using Sigma-Aldrich dialysis membranes with a molecular weight cutoff of ∼14,000 Da. After the dialysis, the suspension was first sonicated for 10 min using a 13-mm-wide titanium probe with an output of 70% (Vibra-Cell VC 750, Sonics), followed by a centrifugation for 10 min at 4000, g. The deagglomerated dispersion was diluted to 0.001 w/w% with MilliQ water and shaken thoroughly.

### Preparation of B-CNC

B-CNCs were prepared from commercially available coconut gel cubes (Chaokoh, Thailand). The coconut cubes (with a size of ∼1 × 1 × 1 cm^3^) were pretreated by first washing for three times with 2 dm^3^ of deionized water, followed by stirring in 2 dm^3^ of a 0.1 M sodium hydroxide solution for 48 h and finally washing with deionized water until the pH stabilized at ∼7. The hydrolysis was performed by soaking 100 g of the pretreated coconut cubes in sulfuric acid with a concentration of 40 w/w% at 80 °C for 4 h. The hydrolysed materials were washed twice with deionized water, collected by centrifugation and dialysed for 5 days against deionized water using Sigma-Aldrich dialysis membranes with a molecular weight cutoff of ∼14,000 Da. After the dialysis, the suspension was first sonicated for 10 min using a 13-mm-wide titanium probe (Vibra-Cell VC 750, Sonics), at an output power of 70%, followed by centrifugation for 60 min at 4000, g. The deagglomerated dispersion was diluted to 0.001 w/w% with MilliQ water and shaken thoroughly.

### Surface charge determination

The surface charge of W-CNC and B-CNC was determined with polyelectrolyte titration using a Stabino Particle Charge Mapping system (Microtrac Europe GmbH, Germany). The nanocellulose dispersions were diluted in MilliQ water and then titrated with a 0.001 N polydiallyl dimethyl ammonium chloride solution. An average charge density was obtained from three or more measurements.

### AFM and PF-QNM measurements

A droplet of 0.001 w/w% solution of different types of cellulose was deposited on chemically modified mica with (3-Aminopropyl)triethoxysilane following a protocol described in ref. 41. AFM and PF-QNM measurements were performed by using a MultiMode VIII Scanning Probe Microscope (Bruker, USA) covered with an acoustic hood to minimize vibrational noise. AFM images were acquired continuously in the tapping mode under ambient conditions using commercial cantilevers (Bruker). In order to perform PF-QNM measurements on all cellulose nanoparticles using the same substrate and under identical environmental and AFM tip conditions, small aliquots of 0.001 w/w% dispersions of W-CNF, W-CNC and B-CNC were mixed in proportion 1:1:1 before deposition on mica surfaces. The AFM cantilevers (Bruker) were calibrated on the calibration samples (Bruker)—typically low-density polyethylene and polystyrene—covering the following ranges of Young's moduli: from 100 MPa to 2 GPa (for low-density polyethylene) and from 1 to 20 GPa (for polystyrene). The measurements were performed on small indentation depths to avoid any artefacts of substrate. The analysis of the elastic modulus was performed using the Nanoscope Analysis software and calculated according the DMT model.

### Transmission electron microscopy

TEM imaging was carried out on carbon-coated copper grids that were glow-discharged for 45 s (Emitech K100X, GB) directly before sample fixation. Sample grid preparation for negative staining was as follows: 5 μl of sample dispersion for 1 min, 5 μl of 2% uranyl acetate for 1 s and again 5 μl of 2% uranyl acetate for 15 s to achieve a noncrystalline film of stain embedding the fibres. Following each step, the excess moisture was drained along the periphery using a piece of filter paper. Dried grids were examined using TEM (FEI, model Morgagni, NL) operated at 100 kV.

### Cryo-SEM

Sample aliquots (3.5 μl) were applied to glow-discharged, carbon-coated Cu-grids for 1 min, blotted with filter paper along the periphery and plunge-frozen in liquid ethane. Vitrified specimens were then transferred and mounted under liquid nitrogen on a self-made grid holder and finally transferred under liquid nitrogen into a precooled (−120 °C) freeze-fracturing system BAF 060 (Bal-Tec/Leica, Vienna). For freeze-drying the samples were warmed up in 5 °C increments every 15 min until −80 °C was reached at 10^−7^ mbar. Coating was performed with 1.5 nm tungsten at 45° followed by 1.5 nm under continuous elevation angle changes from 45° to 90° and back to 45°. Cryo-SEM was performed in a field emission SEM Leo Gemini 1530 (Carl Zeiss, Germany) equipped with a cold stage to maintain the specimen temperature at −110 °C (VCT Cryostage, Bal-Tec/Leica). Signals from the SE-inlens detector (acceleration voltage 5 kV) were used for image formation. Only the contrast and brightness of the pictures were adjusted.

### Tracking of the TEMPO-oxidized W-CNFs

The coordinates of the CNFs (W-CNF) were obtained using an in-house programme written in MATLAB. Each tracked CNF can be represented by its contour—a sequence of points connected with straight segments that are positioned along the fibril bright ridge on an AFM image. All contours acquired in this study have a constant distance between projections of these points on the image plane, which is the step size *s*=2.9 nm. This way of tracking is similar to the procedure we previously applied for amyloid fibril systems^33^,^40^ with one particular addition. Owing to significant directional variation of W-CNF contours (low curvature along rigid segments, high curvature in vicinities of kinks) we employed a concept of masks that define kink areas. The affinity of a contour to bend is different depending on whether points are inside or outside the mask area; in other words, it allows contours to have heterogeneous stiffness (large stiffness along rigid segments and low stiffness in vicinities of kinks). This is essential for the tracking algorithm to correctly follow the actual features of nanocellulose fibrils. The position of these masks for each object and the initial contours were initialized manually, the latter with the auxiliary help of the A* pathfinding algorithm^68^. To obtain subpixel accuracy, contours were deformed and fitted precisely to the cellulose fibrils middle lines using the slightly modified Open Active Contours algorithm^69^,^70^. All statistical information was acquired from high-resolution AFM images with spatial dimensions of 15 × 15 μm and 5,120 × 5,120 pixels. In total, data on 2,380 fibrils from W-CNF were extracted to reach sufficient statistical significance. All data-processing methods used in this work such as the calculation of the kink angle, length and height distributions, persistence length evaluation and plotting fibril height, profiles were performed in the same software.

## Additional information

**How to cite this article:** Usov, I. *et al*. Understanding nanocellulose chirality and structure–properties relationship at the single fibril level. *Nat. Commun.* 6:7564 doi: 10.1038/ncomms8564 (2015).

## Supplementary Material

Supplementary Information Supplementary Figures 1-8
