# Supplementary material for: Understanding nanocellulose chirality and structure–properties relationship at the single fibril level
Source: Nat Commun. 2015 Jun 25;6:7564. doi: 10.1038/ncomms8564 (PMC4491835; doi:10.1038/ncomms8564)
Supplement: Supplementary Information — Supplementary Figures 1-8 [file ncomms8564-s1.pdf]

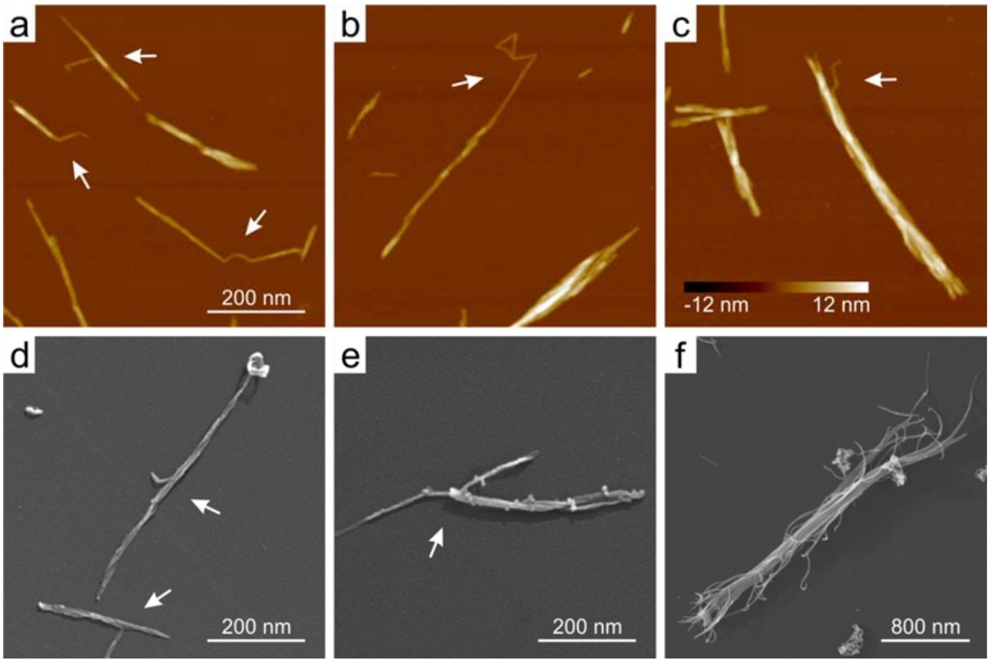

Supplementary Figure 1. Cellulose nanofibrils with kinks and/or splitting events that are pointed by white arrows in the images of the W-CNC, indicating that the hydrolysis was not fully completed. The images were acquired *via* (**a-c**) AFM and (**d-f**) Cryo-SEM. The scale bar in panel **a** and the color bar in panel **c** apply to all AFM images **a-c**.

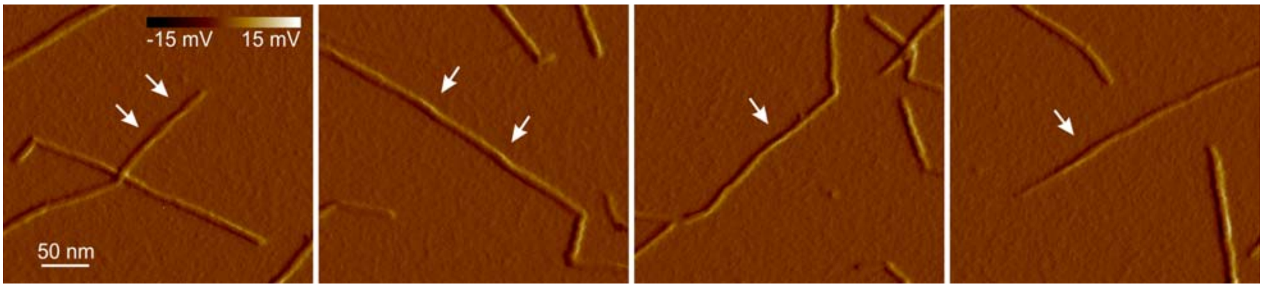

Supplementary Figure 2. Image data from the amplitude channel of AFM scanning showing W-CNF with observable right-handed twisting. The scale and color bars in the left panel apply to all AFM amplitude images.

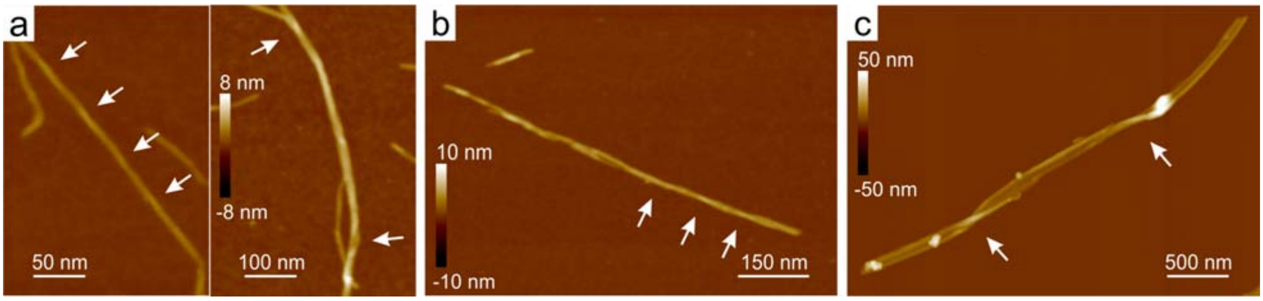

Supplementary Figure 3. Corresponding AFM height maps for the panels **a**, **d**, **g** of the Figure 2 in the main text, with the white arrows reproduced in the same positions.

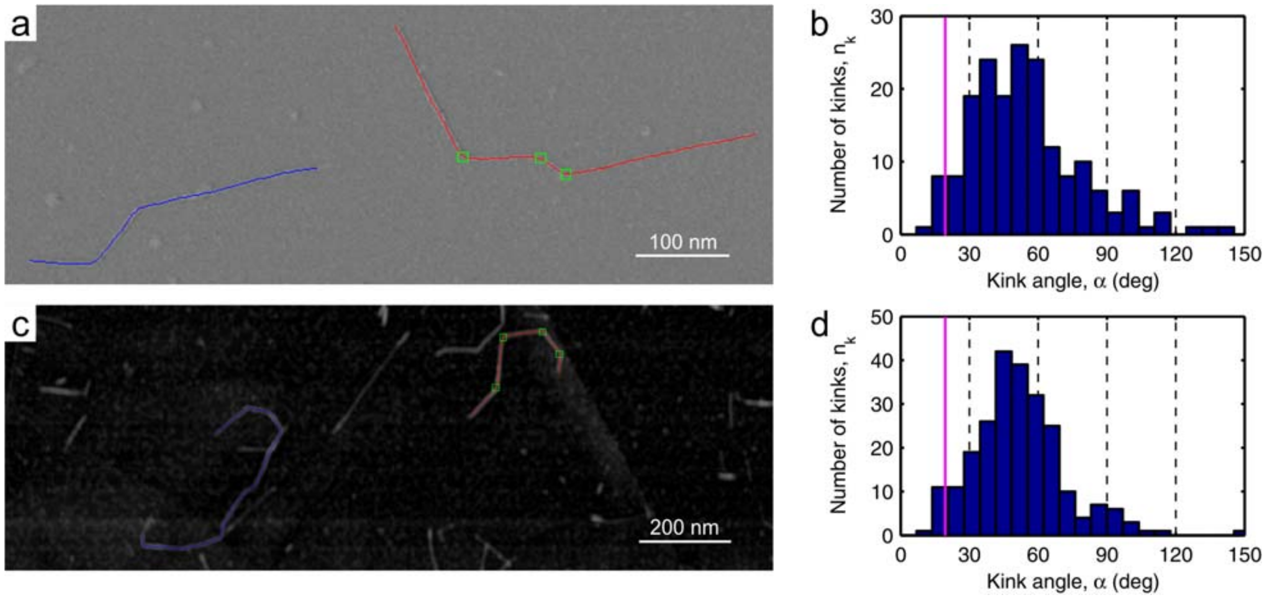

Supplementary Figure 4. An example of (**a**) Cryo-SEM image and (**c**) AFM on graphite with traced W-CNF and manually detected kinks. The corresponding kink angle distributions (**b** and **d**) for the corresponding samples (**a** and **c**).

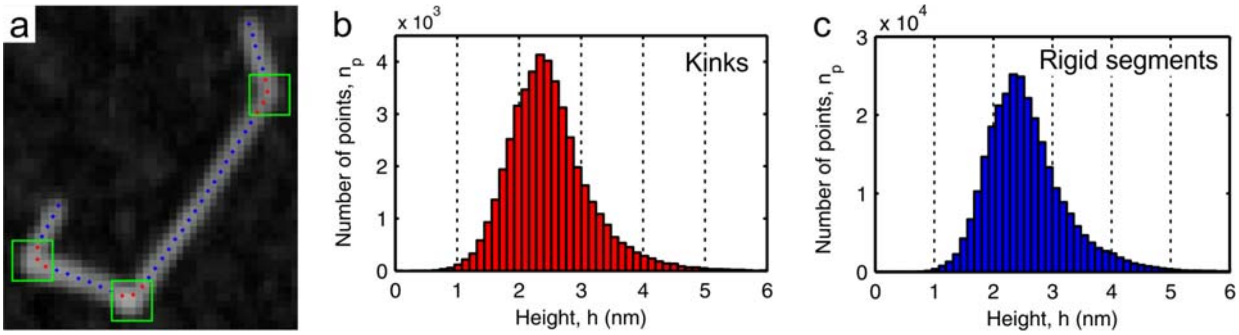

Supplementary Figure 5. A comparison between height distributions of contour points which are located in the vicinities of kinks and along stiff segments in W-CNF. (**a**) Example of the fibril from W-CNF and a corresponding contour with mask elements (green boxes), defining the kink areas. (**b**) The height distribution of all points inside regions of mask elements (red points). (**c**) The height distribution of all points outside regions of mask elements (blue points). Overall, these distributions do not differ from each other.

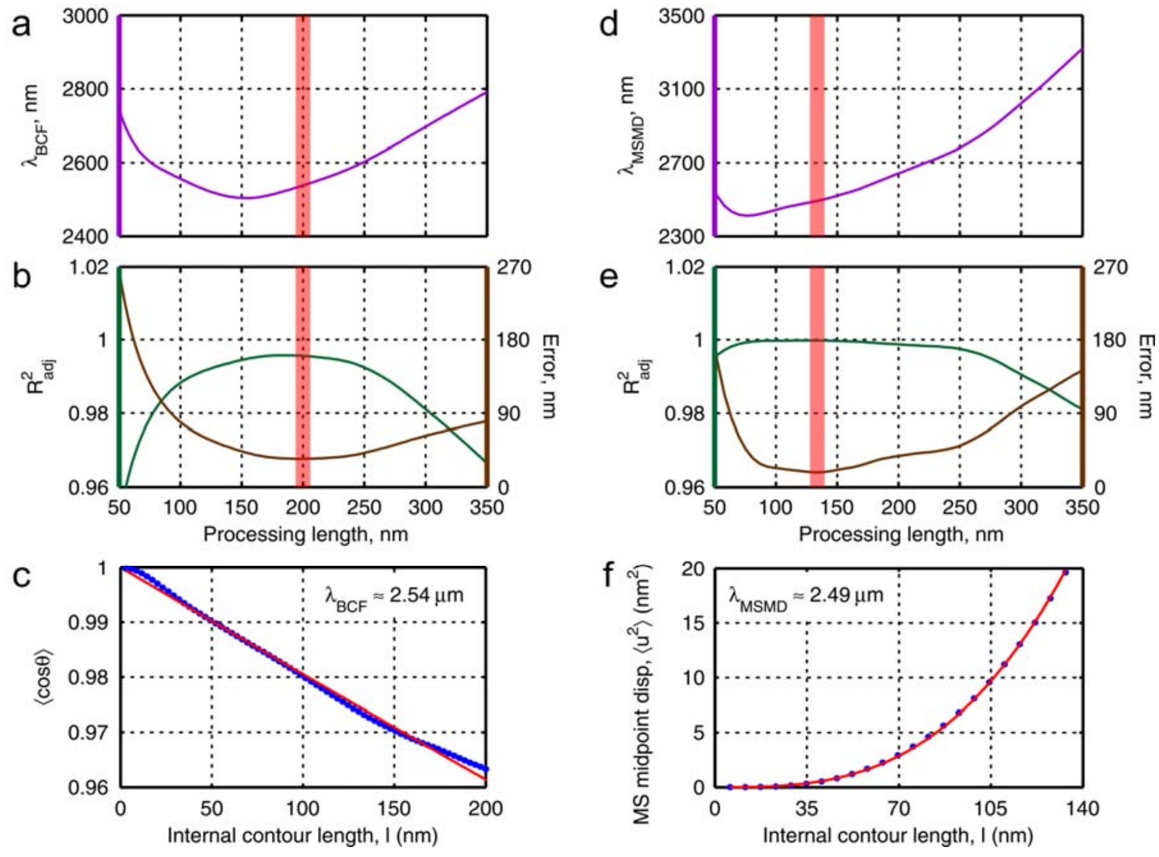

Supplementary Figure 6. The persistence length estimation of the rigid segments derived from W-CNF tracked contours *via* (**a-c**) the bond correlation function method, and (**d-f**) the mean-squared midpoint displacement method. (**a, d**) The persistence length values (λ_{BCF} and λ_{MSMD} respectively) plotted *versus* the processing length. (**b, e**) Adjusted coefficient of determination (goodness of fit) R^2_{adj} and fitting error *versus* the processing length. Horizontal axes from the panels **b** and **e** correspond also to the panels **a** and **d** respectively. The red vertical lines correspond to the processing length 200 nm for the bond correlation function and 135 nm for the mean-squared midpoint displacement methods, at which the R^2_{adj} is maximal and the fitting error is minimal. (**c, f**) The best fits for the bond correlation function and the mean-squared midpoint displacement respectively *versus* the internal contour length, truncated at the optimal processing length. The resulting persistence lengths are $\lambda_{\mathrm{BCF}} = 2.54$ μm and $\lambda_{\mathrm{MSMD}} = 2.49$ μm.

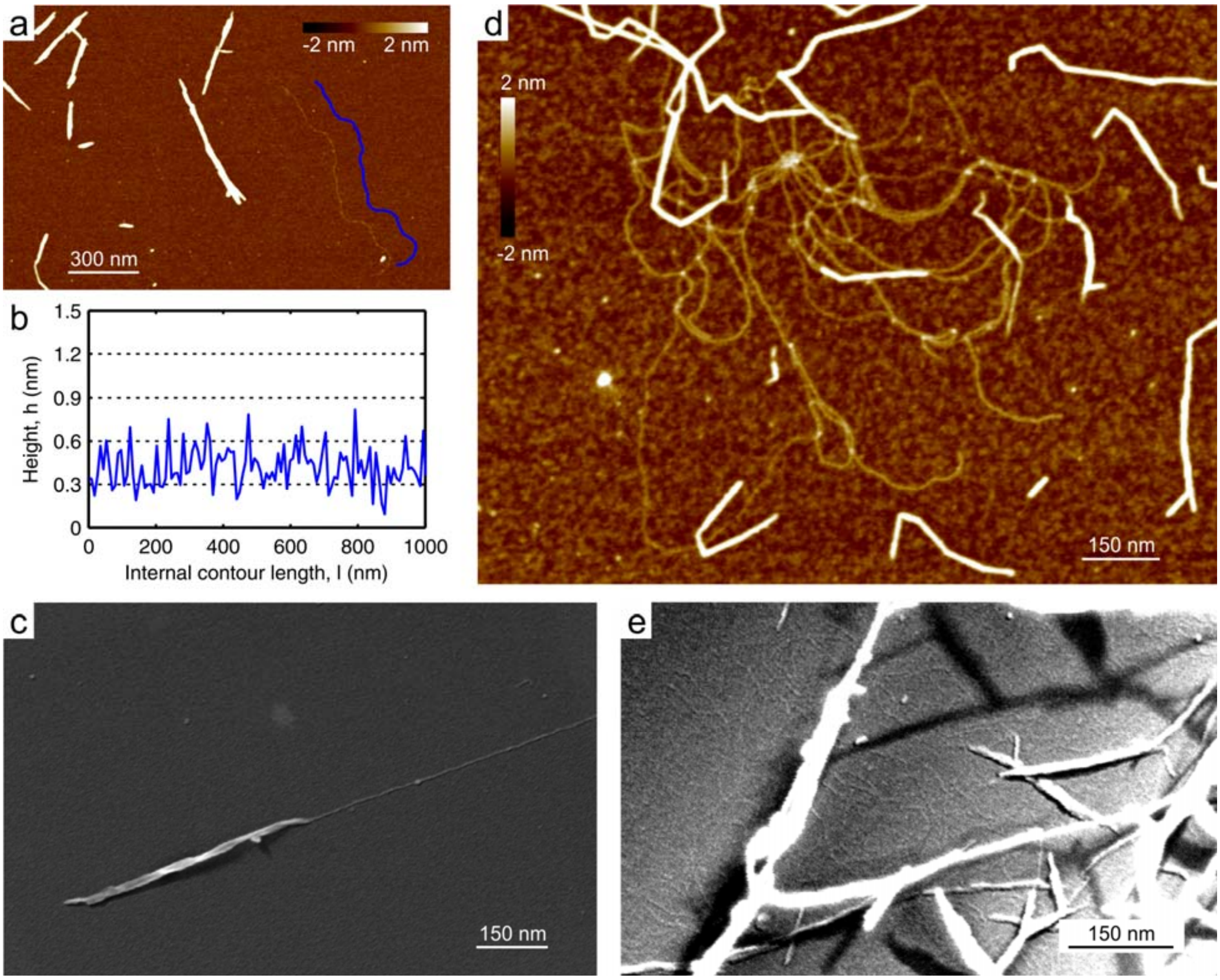

Supplementary Figure 7. (**a**) Observation of a single cellulose polymer chain in AFM image of the W-CNC. The blue line is a representation of the cellulose chain tracked contour, which is shifted for better visualization. (**b**) Height profile along the contour from the panel **a** with the average height value $\langle h \rangle \cong 0.45$ nm. (**c**) Observation of, possibly, a single cellulose polymer chain in Cryo-SEM image of the W-CNC. (**d**) AFM image of W-CNF, depicting single and 2×2 cellulose polymer chains forming a remarkable network with a well-defined entanglement center. (**e**) Cryo-SEM image with a high contrast of W-CNC, showing similar network in the background.

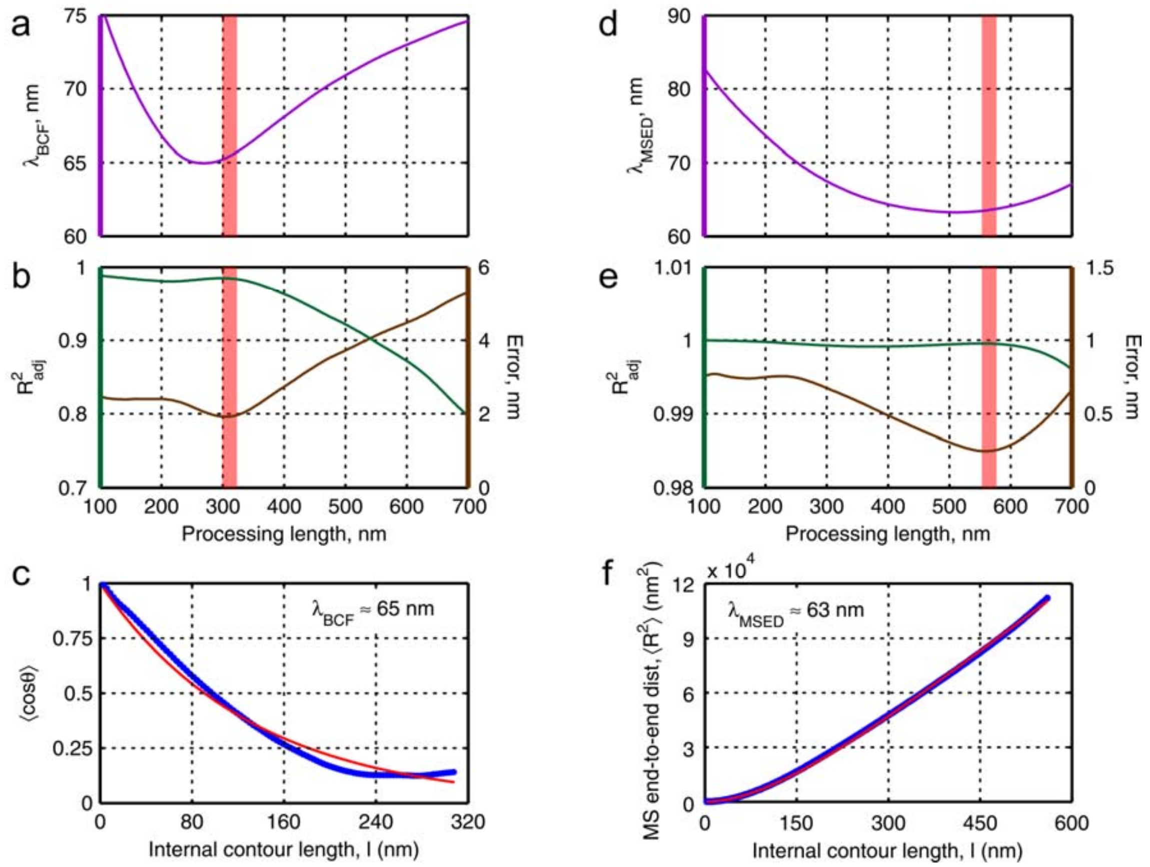

Supplementary Figure 8. The persistence length estimation of single cellulose polymer chain contours *via* (**a-c**) the bond correlation function method and (**d-f**) the mean-squared end-to-end distance method. (**a, d**) The persistence length values (λ_{BCF} and λ_{MSED} respectively) plotted *versus* the processing length. (**b, e**) Adjusted coefficient of determination (goodness of fit) R_{adj}^2 and fitting error *versus* the processing length. Horizontal axes from the panels **b** and **e** correspond also to the panels **a** and **d** respectively. The red vertical lines correspond to the processing length 307 nm for the bond correlation function and 560 nm for the mean-squared end-to-end distance methods, at which the R_{adj}^2 is maximal and the fitting error is minimal. (**c, f**) The best fits for the bond correlation function and the mean-squared end-to-end distance respectively *versus* the internal contour length, truncated at the optimal processing length. The resulting persistence lengths are $\lambda_{\mathrm{BCF}} = 65$ nm and $\lambda_{\mathrm{MSED}} = 63$ nm.
